# ESR Essentials: diffusion-weighted MRI—practice recommendations by the European Society for Magnetic Resonance in Medicine and Biology

**DOI:** 10.1007/s00330-025-12033-x

**Published:** 2025-10-02

**Authors:** Marco Palombo, Benedetta Bodini, Francesco Grussu, Denis Le Bihan, Markus Nilsson, Raquel Perez-Lopez, Edwin H. G. Oei, Ivo G. Schoots, Marion Smits, Ileana O. Jelescu

**Affiliations:** 1 Cardiff University Brain Research Imaging Centre (CUBRIC), School of Psychology, Cardiff University, Cardiff, UK; 2 School of Computer Science and Informatics, Cardiff University, Cardiff, UK; 3 Paris Brain Institute, Sorbonne Université, Paris, France; 4 AP-HP, Hôpital Universitaire Pitié-Salpêtrière, Paris, France; 5 Radiomics Group, Vall d’Hebron Institute of Oncology, Barcelona, Spain; 6 NeuroSpin, CEA-Saclay Center, Paris-Saclay University, Gif-sur-Yvette, France; 7 Human Brain Research Center, Kyoto University Graduate School of Medicine, Kyoto, Japan; 8 Department of System Neuroscience, National Institutes for Physiological Sciences, Okazaki, Japan; 9 Brain and Mind Research Center, Nagoya University, Nagoya, Japan; 10 Department of Clinical Sciences Lund, Radiology, Lund University, Lund, Sweden; 11 Department of Radiology and Nuclear Medicine, Erasmus MC, University Medical Center Rotterdam, Rotterdam, The Netherlands; 12 Department of Radiology, Netherlands Cancer Institute—Antoni van Leeuwenhoek Hospital, Amsterdam, The Netherlands; 13 Department of Radiology, Lausanne University Hospital (CHUV), Lausanne, Switzerland; 14 Faculty of Biology and Medicine, University of Lausanne, Lausanne, Switzerland

**Keywords:** Diffusion magnetic resonance imaging, Recommendations, Human body, Human brain, Radiology

## Abstract

**Abstract:**

Diffusion-weighted imaging (DWI) offers critical insights into tissue microstructure through the assessment of water molecule random displacements and plays a central role in the assessment of neoplastic and non-neoplastic diseases. To successfully implement and use DWI in clinical practice, guidelines for acquisition, interpretation of image contrast and of artefacts should be followed, taking the disease process and body part into account. We recommend covering a *b*-value range of 0–1000 s/mm^2^ in the brain (along at least six directions for white matter), and 50–800 s/mm^2^ in the body. Available acquisition acceleration options should be used to reduce repetition time (TR), echo time (TE), and echo-planar imaging (EPI) distortions, while considering the penalty in signal-to-noise ratio (SNR) and image sharpness. DW images and the apparent diffusion coefficient (ADC) map should be read jointly for the clinical interpretation. Areas of slower diffusion are hyperintense on DW images and hypointense on the ADC map, and vice versa. Magnetic susceptibility distortions and signal drop-outs or pile-ups are particularly pronounced at air-tissue or metal-tissue interfaces and may obscure areas of interest or hinder the co-localisation with structural scans. By following these guidelines and recommendations, radiologists and imaging professionals can enhance diagnostic accuracy, reduce variability, and maximise the clinical value of DWI across diverse applications.

**Key Points:**

- *This article provides an overview of DWI principles, clinical applications, potential pitfalls, and emerging advances, alongside expert recommendations for optimal implementation*.
- *We provide key considerations tailored to specific applications (neuro and whole-body imaging), including protocol optimisation, adherence to established guidelines, and quality assurance measures to minimise artefacts and ensure reproducibility*.
- *By following the guidelines and recommendations summarised in this work, radiologists and imaging professionals can enhance diagnostic accuracy, reduce variability, and maximise the clinical value of DWI across diverse applications*.

## Key recommendations

- The sequence most recommended for clinical diffusion MRI is single-shot EPI. Long repetition times TR (≥ 4500 ms) and minimal echo times TE should be used to minimise T_1_- and T_2_-weighting and maximise signal-to-noise ratio (SNR). Consider simultaneous multi-slice imaging to reduce TR and scan time but monitor slice cross-talk artefacts and residual T_1_-weighting. Parallel imaging or segmented EPI read-outs should be used to reduce TE and EPI distortions; trade-offs: SNR loss and extended scan time. For cardiac and liver applications, respiratory/cardiac gating is recommended to improve quality. Increasing the number of averages proportionally to the square root of the b-value is recommended to compensate for SNR loss at high *b*-values (evidence level: high—further research is unlikely to change our confidence in the recommendations).
- The 3–4 directions acquisition and “4-scan trace” may be used to mitigate tissue anisotropy effects in the brain or gradient miscalibration effects. For highly anisotropic tissues (e.g., brain white matter and kidney), if unbiased quantitative ADC is needed, we recommend using a full diffusion tensor model (one *b* = 0 and ³6 non-collinear directions) to fully remove effects of anisotropy. ADC maps should be included in the interpretation to remove T_2_-shine-through. Reporting the diffusion time (if available) is recommended for reproducibility (evidence level: high—further research is unlikely to change our confidence in the recommendations).
- The analysis should be integrated as much as possible into clinical workflows. Readers should be aware of typical DWI artefacts: e.g., geometric distortions may hinder the exact co-localisation of DWI hyper-/hypo-intensities with anatomical features identifiable on structural, high-resolution scans. Distortion/motion correction algorithms can be used to improve DWI alignment. Unprocessed images (e.g., without interpolation) should also be stored if space allows, for the retrospective analysis with advanced image processing tools (evidence level: high—further research is unlikely to change our confidence in the recommendations).

## Introduction

Since its development in the mid-1980s, diffusion-weighted imaging (DWI) has become an indispensable tool in radiology, providing unique insights into tissue microstructure by measuring the random motion of water molecules.

The protocol used for DWI affects the diffusion contrast, image quality, and accuracy. Standardised recommendations are essential to ensure consistent and optimal imaging across different scanners and institutions.

**Fig. 1:**
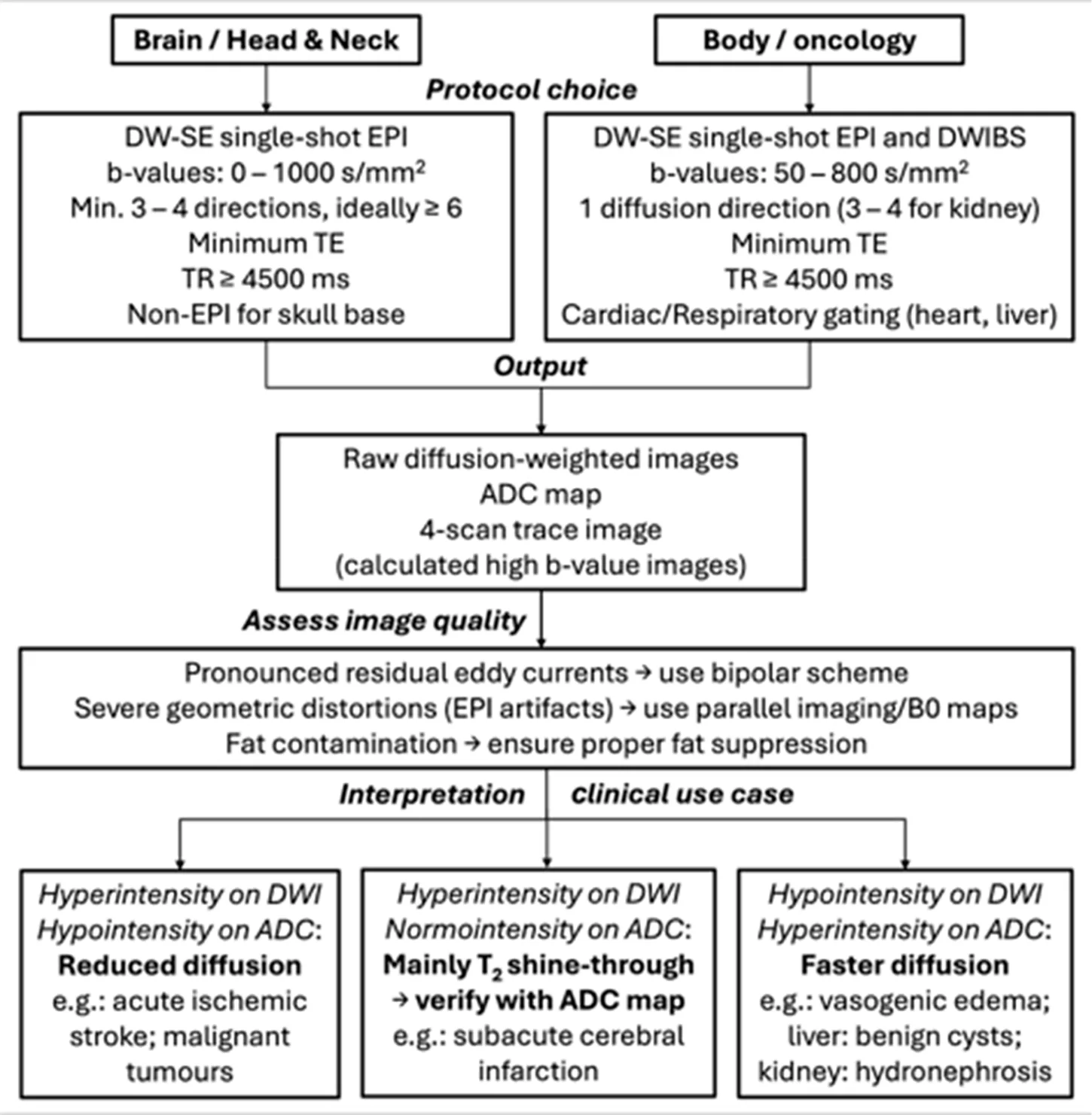
Synthetic flowchart for practical setup and radiological assessment of DWI data in clinical practice. Critical steps include assessment of image quality and artefact mitigation (e.g., eddy current compensation/correction, fat suppression, non-EPI sequences to avoid severe distortion artefacts). For highly anisotropic tissues, employ ≥ 6 diffusion directions and DTI to avoid bias due to residual anisotropy in ADC maps. Cross-validate hyperintense DWI signals with ADC values to distinguish true restriction (low ADC, e.g., in acute ischaemic stroke) from T2-shine-through (high ADC, caused by prolonged T2 relaxation, e.g., in subacute cerebral infarction). In oncology (e.g., prostate or breast), use DWIBS to enhance tumour detectability and optionally combine high *b*-value DWI (e.g., *b* = 1500 s/mm^2^) with ADC maps to assess cellularity. DWI, diffusion-weighted imaging; *b*-value, diffusion weighting; DW-SE, diffusion-weighted spin echo; EPI, echo planar imaging; DWIBS, diffusion-weighted imaging with background body signal suppression; DTI, diffusion tensor imaging; TE, echo time; TR, repetition time; ADC, apparent diffusion coefficient; 4-scan trace image, average of diffusion measurements along 4 different directions

This article provides an overview of DWI for radiologists and imaging professionals, covering its principles, clinical applications, potential pitfalls and emerging advances, with key recommendations and guidelines from experts in DWI of the European Society for Magnetic Resonance in Medicine and Biology (ESMRMB) for proper use and interpretation in clinical practice. A synthetic flowchart of practical recommendations is provided in Fig. 1.

## Principles of DWI

**Fig. 2:**
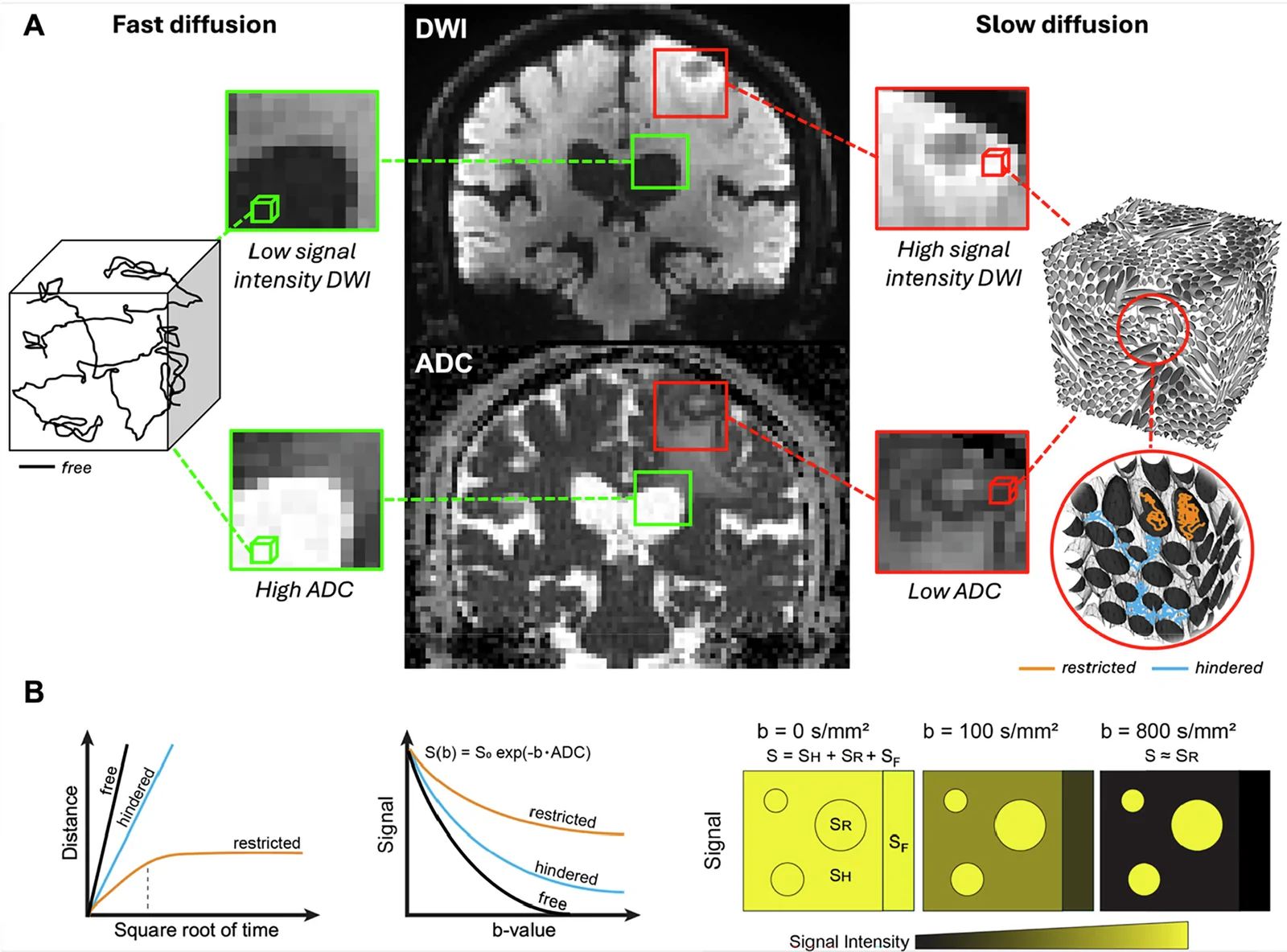
Simplified physical basis of diffusion MRI for an exemplar tumour case. **A** Water molecules undergoing random motion within two representative tissue types (green and red boxes) are shown: fast-diffusing free water (black) in CSF, and slow-diffusing water restricted (orange) and hindered (blue) in tissue packed with cells proliferating. The measured DWI signal intensity reflects the properties of the underlying water diffusion process, showing low values for the fast diffusion and high values for the slow diffusion cases. The corresponding ADC shows the opposite trend. The DWI and ADC images are representative images from a patient with brain metastasis, here used only for illustrative purposes. **B** Water molecules in free and hindered diffusion show a linear increase in distance with the square root of the diffusion time (Gaussian diffusion), with free water (black) covering a longer distance than hindered water (blue) at the same time. Water restricted in the intracellular space exhibits Gaussian diffusion at short timescales (orange) but becomes non-Gaussian (sublinear) at longer timescales as molecules encounter membrane boundaries (dotted line). In DWI, the signal (S) decays exponentially with *b*-value. Free diffusion (black) decays faster than hindered (blue), and both decay faster than restricted (orange). At short timescales and low *b*-value, S combines free (SF), hindered (SH) and restricted (SR) signals. At longer times and higher *b*-values, free and hindered signals dissipate faster, leaving mostly the restricted signal. Restricted water retains more signal at high *b*-values, yielding a lower ADC. Adapted from Mendez, Fang, Meriwether et al (2022). Diffusion breast MRI: current standard and emerging techniques. Front Oncol 12:844790. https://doi.org/10.3389/fonc.2022.844790

Brownian motion was discovered under a microscope as the spontaneous, random movement of pollen grains suspended in water. Later, Einstein demonstrated that this phenomenon resulted from the invisible collisions of water molecules with the pollen, providing a crucial link between macroscopic observations and microscopic physics. Diffusion MRI is built on this view: diffusion-weighted images (DWIs) at the *millimetre* scale reflect the underlying Brownian motion of water molecules in tissues, which, in turn, reveals the many obstacles they encounter at the *microscopic* scale, hindering their displacements during the measurement (diffusion) time, typically 50-80 ms. While in a cyst or vasogenic oedema (or cerebrospinal fluid (CSF)), water diffusive displacements are somewhat free, those displacements become hindered or restricted in tissues packed with proliferating cells, such as in malignant tumours, swelling, or cytotoxic oedema associated with acute brain ischaemia, which reduces the measured apparent diffusion coefficient (Fig. 2 illustrates these concepts using tumours as an example). Hence, the result of diffusion MRI measurements is called the apparent diffusion coefficient (ADC) to emphasise that it is not the genuine (free) diffusion coefficient of water [1]. The success of diffusion MRI relies, indeed, on this exquisite ADC sensitivity to the underlying tissue microstructure (i.e., the tissue structure at the microscopic scale), providing, to some extent, a kind of virtual biopsy.

**Fig. 3:**
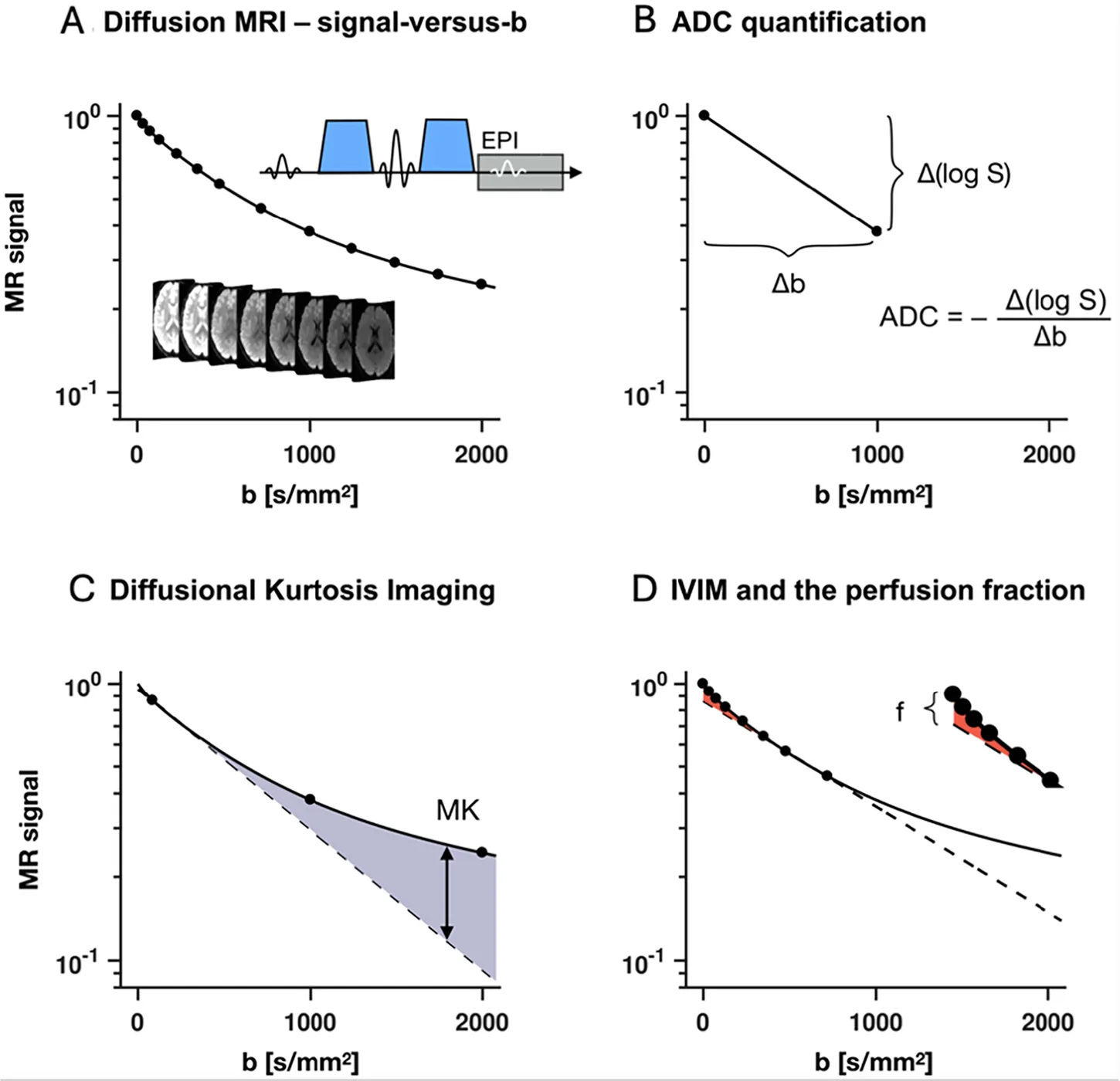
Overview of diffusion-weighted MRI methods. **A** Conventional diffusion encoding varies the diffusion-encoding strength (so-called *b*-value) by increasing the amplitudes of the encoding gradients (blue in panel **A**). Higher *b*-values result in stronger signal attenuation and a change in image contrast. **B** The apparent diffusion coefficient (ADC) is assessed from the slope of the logarithm of the signal attenuation curve at a low *b*-value, often 1000 s/mm^2^. **C** Diffusional kurtosis imaging utilises higher *b*-values and assesses the departure of the signal curve from mono-exponential attenuation (shadowed region between dashed and solid line). **D** The intravoxel incoherent motion approach estimates the perfusion fraction (f) from the small overshoot of the signal at low *b*-values (red region in inset). Adapted from Nilsson, Englund, Szczepankiewicz et al (2018) Imaging brain tumour microstructure. Neuroimage 182:232–250. https://doi.org/10.1016/j.neuroimage.2018.04.075

Diffusion effects appear in MRI signals when the motion of molecules occurs within inhomogeneous magnetic fields (so-called gradients), as molecules experience different field strengths along their trajectories, resulting in so-called phase shifts. However, the standard magnetic field gradient pulses used for MRI are not strong enough. Hence, for diffusion MRI, dedicated gradient pulses are added within MRI sequences (typically a T_2_-weighted spin-echo sequence). Due to the random nature of the diffusive motion, the phase shifts of billions of molecules add up in an incoherent manner, resulting in an overall attenuation of the amplitude of the MRI (echo) signal. This attenuation depends on both the history of the molecular displacements, hence the ADC in each voxel (high diffusion results in larger displacements and greater attenuation), and the intensity and time profile of the gradient pulses (the diffusion-weighting). The degree of diffusion-sensitisation provided by this gradient profile is quantified by the so-called “*b*-value” [1]. The *b*-value is to diffusion-weighting what the echo time (TE) is to T_2_-weighting: high *b*-values result in stronger diffusion-related signal attenuation and stronger diffusion contrast (Fig. 3A). Note that in the absence of diffusion gradients, the effective *b*-value is never exactly zero, due to the contribution of the imaging gradients.

In DWI, voxels exhibiting high diffusion are dark while those exhibiting low diffusion are bright (Fig. 2). To obtain quantitative ADC images, two sets of DWIs are acquired with the same TE but different *b*-values (e.g., 0 s/mm^2^ and 1000 s/mm^2^). The ADC is derived voxel-wise from the ratio of the two signals (Fig. 3B). ADC maps have opposite contrast to DWIs (high diffusion voxels are bright—Fig. 2). In a similar way to TE values that are chosen to maximise T_2_ contrast while maintaining sufficient signal-to-noise ratio (SNR), optimal pairs of *b*-values depend on the ADC of the tissue of interest, often around 0–800 s/mm^2^ in the body and 0–1000 s/mm^2^ in the brain. However, larger *b*-values (2000–3000 s/mm^2^) might provide more specific information on tissue microstructure, as the effects of restriction and hindrance to diffusion from cell membranes or fibres become more prominent. Quantification of such “non-Gaussian” effects requires the use of more advanced markers than the ADC (Fig. 3C).

## Technical recommendations

### Sequence design and optimisation

Diffusion-weighted spin echo single-shot echo-planar imaging (EPI) is recommended for most clinical DWI, but alternative readouts may be needed in regions with strong distortions. Due to the spin echo scheme, DWIs also entangle T_1_ and T_2_-weighting.

T_1_-weighting can be controlled through the repetition time (TR). In DWI, long TR is generally needed for multi-slice acquisition, which then also allows for substantial T_1_-recovery, increasing SNR and minimising T_1_-effects. Typically, we recommend a TR value of 4500 ms or higher (3–5 times the expected tissue T_1_) to minimise T_1_-weighting. We recommend using simultaneous Multi-Slice Imaging (also known as multi-band imaging) to reduce the minimum TR and scan time by acquiring slices concurrently. However, it may lower SNR (due to incomplete T_1_-recovery) and introduce T_1_-weighting.

T_2_-weighting of DWIs increases with the TE. Long TE may be required to achieve high *b*-values with clinical scanners, due to limitations in the gradient strength (i.e., longer gradient pulses must be used). Long TE improves T_2_-contrast between tissues and within-voxel components (e.g., intracellular water, oedema, lumens, vessels) [2], but reduces SNR due to T_2_-decay. In practice, we recommend using the (same) minimum TE compatible with the highest targeted *b*-value for all acquired *b*-values.

Two diffusion encoding schemes may be available: monopolar (offering shorter TE, higher SNR) and bipolar (reducing eddy current effects). Choice depends on scanner performance: prefer monopolar with good eddy current compensation, bipolar otherwise.

Regarding the number of diffusion directions, one *b* = 0 and 3–4 DWIs acquired along different directions enable ADC computation while mitigating slight tissue anisotropy. For highly anisotropic tissues (e.g. in brain white matter, kidney), due to imperfect cancellation of cross-terms from imaging gradients, a full diffusion tensor imaging (DTI) framework is recommended [3], with one *b* = 0 image and at least six DWIs across non-collinear directions. Whenever possible, the “diffusion time”, often unspecified by manufacturers, should be reported since it influences ADC values and cross-site reproducibility.

Due to DWI’s inherently low SNR, careful parameter optimisation is crucial. Key factors include resolution, maximum *b*-value, acceleration methods (parallel imaging, partial Fourier, multiband), and minimum TE. To maintain optimal SNR across varying *b*-values, we recommend increasing the number of averages (NA) proportionally to the square root of the *b*-value, compensating for the inherent SNR decline at higher *b*-values. We recommend parallel imaging (e.g., SENSE and GRAPPA) to reduce TE and EPI spatial distortions; however, it lowers SNR. Similarly, higher bandwidth shortens TE but also decreases SNR. For non-brain applications, consider respiratory/cardiac gating (improves quality but increases scan time and TR’s variability) [4]. Pre-study optimisation tests in healthy volunteers are recommended to balance scan time and image quality.

Finally, efficient and homogeneous fat suppression is key to avoid obscuring structures of interest with the strong fat signal (due to its short T_1_ and low diffusion coefficient) and avoiding ghosting from the subcutaneous fat.

### Parameter maps and derived images

**Fig. 4:**
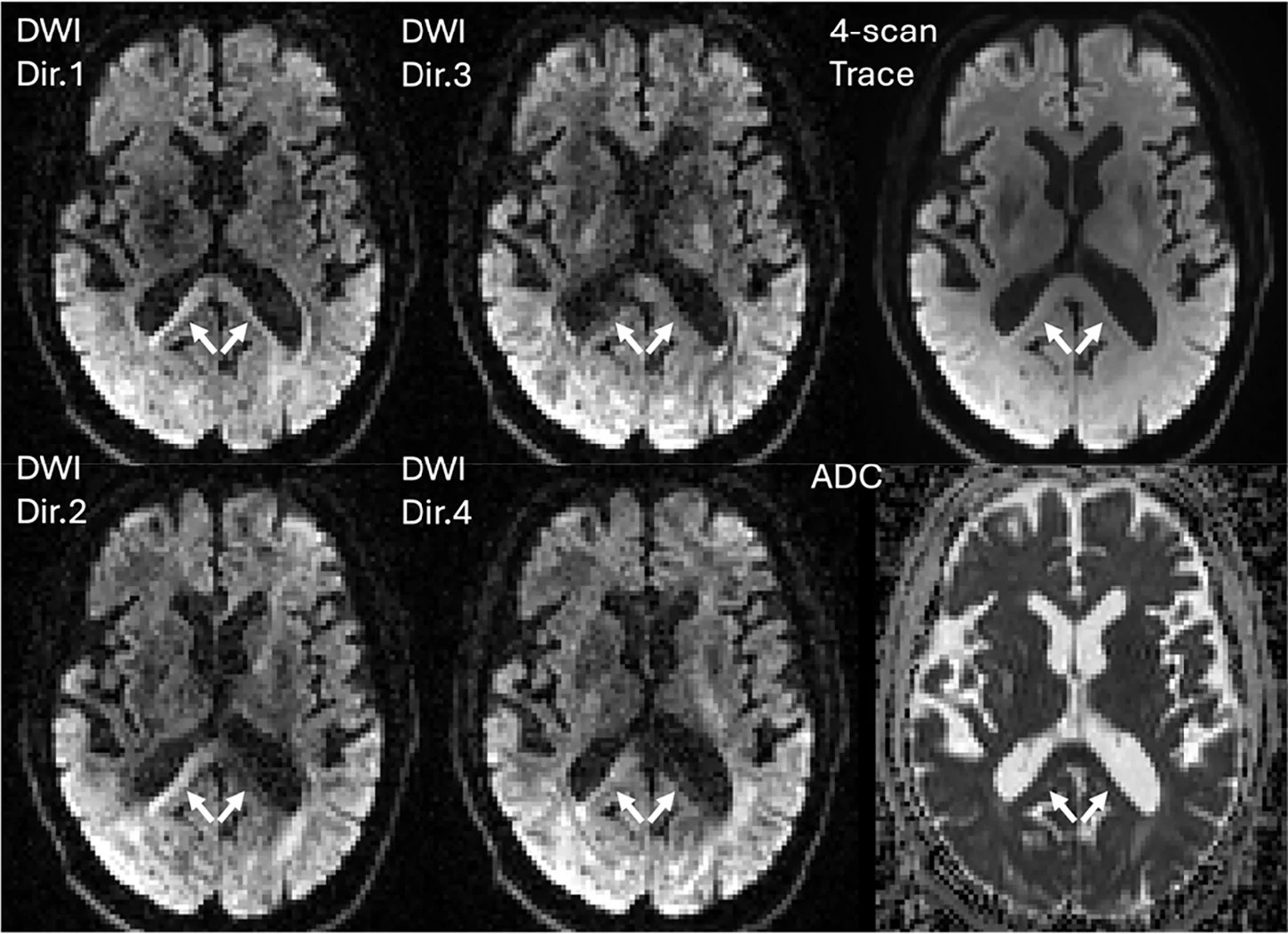
Exemplar 4-scan trace DWI data. DWI images are acquired at a given *b*-value (here 1000 s/mm^2^) with diffusion gradients oriented along four different directions (DWI Dir.1-4). The mean DWI image (4-scan trace) is then computed by averaging the DWI images along the four directions, and an ADC map is estimated from it. Arrows indicate signal changes due to tissue anisotropy, mitigated in the 4-scan trace and the corresponding ADC map

The default calculations performed directly at the scanner console include the ADC maps and the so-called “4-scan trace” image (an average of diffusion measurements along four different directions, for each *b*-value). We recommend the ADC map computed from three to four DWIs acquired along different directions and/or the 4-scan trace image, as a practical compromise between speed and reduced anisotropy bias (Fig. 4), though residual errors may persist in highly anisotropic tissues due to cross-terms between imaging and diffusion gradient pulses, which cannot be neglected [5]. In such cases, a full DTI protocol is preferred to properly quantify anisotropy and ensure unbiased ADC calculations. The choice depends on clinical priorities: efficiency versus precision. While an ADC derived from a 3- or 4-scan trace is likely acceptable in most clinical applications, it remains an approximation that can become problematic if quantitative ADC thresholds are used to classify lesions, or if data are compared/aggregated across different sites and vendors, which requires standardisation [6].

The DTI framework [3] estimates the ADC in any given encoding direction, which can present large variations: e.g., in white matter, diffusivity along the fibres is much faster than perpendicular to them due to the higher restriction and tortuosity in the latter case (Fig. 4). The most common quantitative parameters derived from DTI are the mean diffusivity (MD)—the average ADC over all directions (similar to the 4-scan trace ADC)—and the fractional anisotropy (FA)—a normalised measure (scaled 0–1) of how large the difference (or variance) is between the ADC in different directions (FA = 0 for isotropic tissue, e.g., CSF; FA = 1 for highly anisotropic tissue, e.g., the internal capsule). DTI reconstructions are increasingly available online at the console, and they are the first step towards a tractography reconstruction (see “Beyond the ADC” section).

To enhance the contrast-to-noise ratio (CNR) and aid the visual identification of abnormal areas, a synthetic DWI referred to as “calculated *b*-value” image is sometimes computed, extrapolating a specific *b*-value higher than those acquired (e.g., *b* = 1500 s/mm²) from *b* = 0 and ADC maps. It always correlates with acquired (non-synthetic) images and is not entirely accurate because it cannot predict the signal behaviour from non-Gaussian effects, which could lead to misinterpretation of some lesion content [7].

### Image artefacts

DWI and ADC mapping are associated with three main types of artefacts: T_2_-shine-through, geometric distortions and signal pile-up, and motion and eddy current distortions that result in misalignments across DWI images and potential blurring. T_2_-shine-through occurs when a diffusion-weighted signal appears hyperintense, not due to reduced diffusion but because the T_2_-weighted signal obtained without diffusion encoding is strong. Indeed, DWI intensity is the product of the T_2_-weighted image intensity and the attenuation caused by diffusion weighting. A hyperintense DWI signal can result from either or both components being high. Geometric distortions can lead to signal pile-up, where the signal from multiple voxels is compressed into fewer ones, artificially increasing the DWI signal. Like T_2_-shine-through, this can create a hyperintense appearance unrelated to actual diffusion changes. Geometric distortions and signal pileups arise in EPI sequences due to magnetic field inhomogeneities, typically caused by susceptibility differences between air and tissue. First, good prospective B_0_ shimming is therefore critical for DWI quality. Furthermore, these distortions can be mitigated using parallel imaging, segmented readout strategies, or non-EPI readouts such as HASTE (the single-shot form of the widely used RARE), or post-processing techniques based on B_0_ field mapping. Finally, strong magnetic field gradients used in DWI generate eddy currents in the MRI scanner. These induce slight image distortions, misaligning DWI scans with morphological sequences. These artefacts can be addressed in post-processing: T_2_-shine-through is effectively eliminated in the ADC map, geometric distortions can be reduced by dedicated algorithms, and motion and eddy currents can be corrected using yet another set of dedicated algorithms. Many MRI vendors incorporate some or all of these corrections into their diffusion imaging packages. In research settings, these steps can also be performed using open-source tools, e.g., FSL, MRtrix3, and DiPy.

## Clinical recommendations

### Neuro and head and neck

**Fig. 5:**
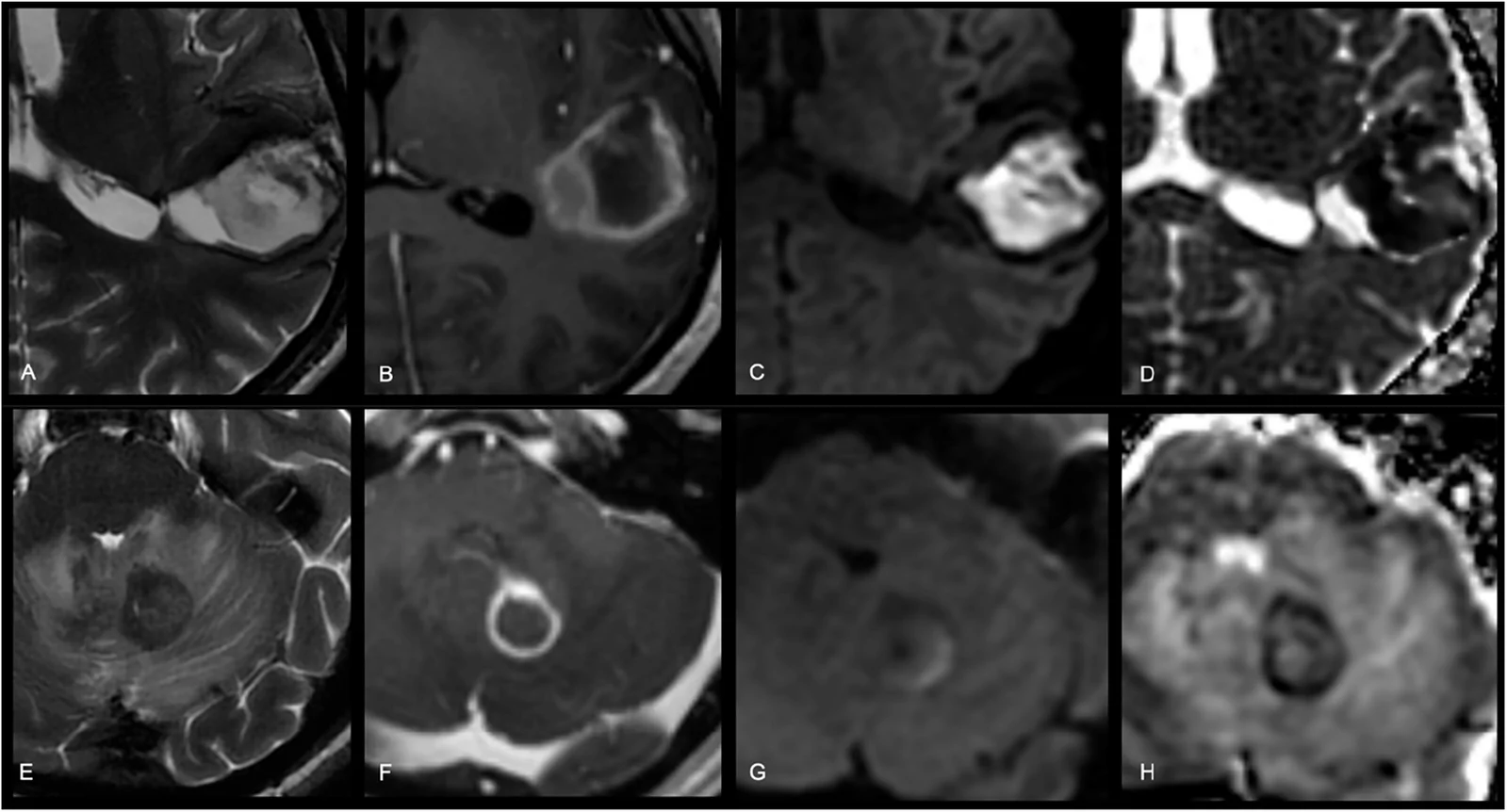
Pitfalls in the interpretation of diffusion restriction in ring-enhancing brain lesions. Top row (A-D) shows a heterogeneous T2-hyperintense lesion in the left parietal lobe (**A**) with the appearance of ring-enhancement on post-contrast T1-weighted imaging (**B**), central high signal on DWI (**C**), with corresponding low ADC (**D**) indicating restricted diffusion. While diffusion restriction suggests an abscess, the diagnosis was a lobar haemorrhage. Bottom row (**E**–**H**) shows a T2-hypointense lesion in the left cerebellum (**E**) with ring-enhancement on post-contrast T1-weighted imaging (**F**). The central portion of the lesion shows an intermediate DWI signal (**G**) and ADC (**H**). Despite a lack of diffusion restriction, the diagnosis was a toxoplasmosis abscess. Especially in abscesses from atypical organisms (e.g., toxoplasmosis and tuberculosis), diffusion is not always restricted

We recommend using DWI for detecting acute brain ischaemia and differentiating the infarct core from the penumbra. DWI findings are highly dependent on time post-infarction: after an initial dramatic ADC drop, ADC pseudo-normalisation [8] occurs after ~1 week, due to the combination of both cytotoxic and vasogenic oedema as well as cell membrane breakdown. Subsequently, ADC and T_2_ values continue to increase, the latter resulting in T_2_-shine-through. DWI is also indispensable for differentiating between a necrotic tumour and an abscess. While accuracy is very high (> 95% [9]), it is not perfect (Fig. 5); we recommend always correlating with the clinical context. DWI has revolutionised cholesteatoma surveillance, reducing the need for second-look surgery. We recommend non-EPI imaging to avoid EPI-related artefacts at the skull base [10].

### Body/oncology

DWI plays a growing role across several cancer types [11], with particularly well-established applications in breast and prostate cancer. In breast cancer, DWI supports the non-invasive differentiation of benign from malignant lesions [12, 13], as well as the distinction between metastatic and non-metastatic lymph nodes, contributing to accurate staging and treatment planning [14, 15]. In prostate cancer, DWI is a cornerstone of multiparametric MRI and a central element of the PI-RADS assessment system. It plays a key role in lesion detection and risk stratification, particularly in the peripheral zone, where DWI is the dominant sequence [16, 17]. We recommend using ADC maps and high *b*-value images to enhance lesion conspicuity and improve confidence in distinguishing clinically significant cancer from benign conditions such as prostatitis or hyperplasia. In liver cancer, the 2024 LI-RADS update [18] recognises restricted diffusion as an ancillary feature to help identify viable tumours in equivocal cases, improving early detection after local-regional therapy. Generally, we recommend using ADC maps in combination with high *b*-values for improved lesion conspicuity.

**Fig. 6:**
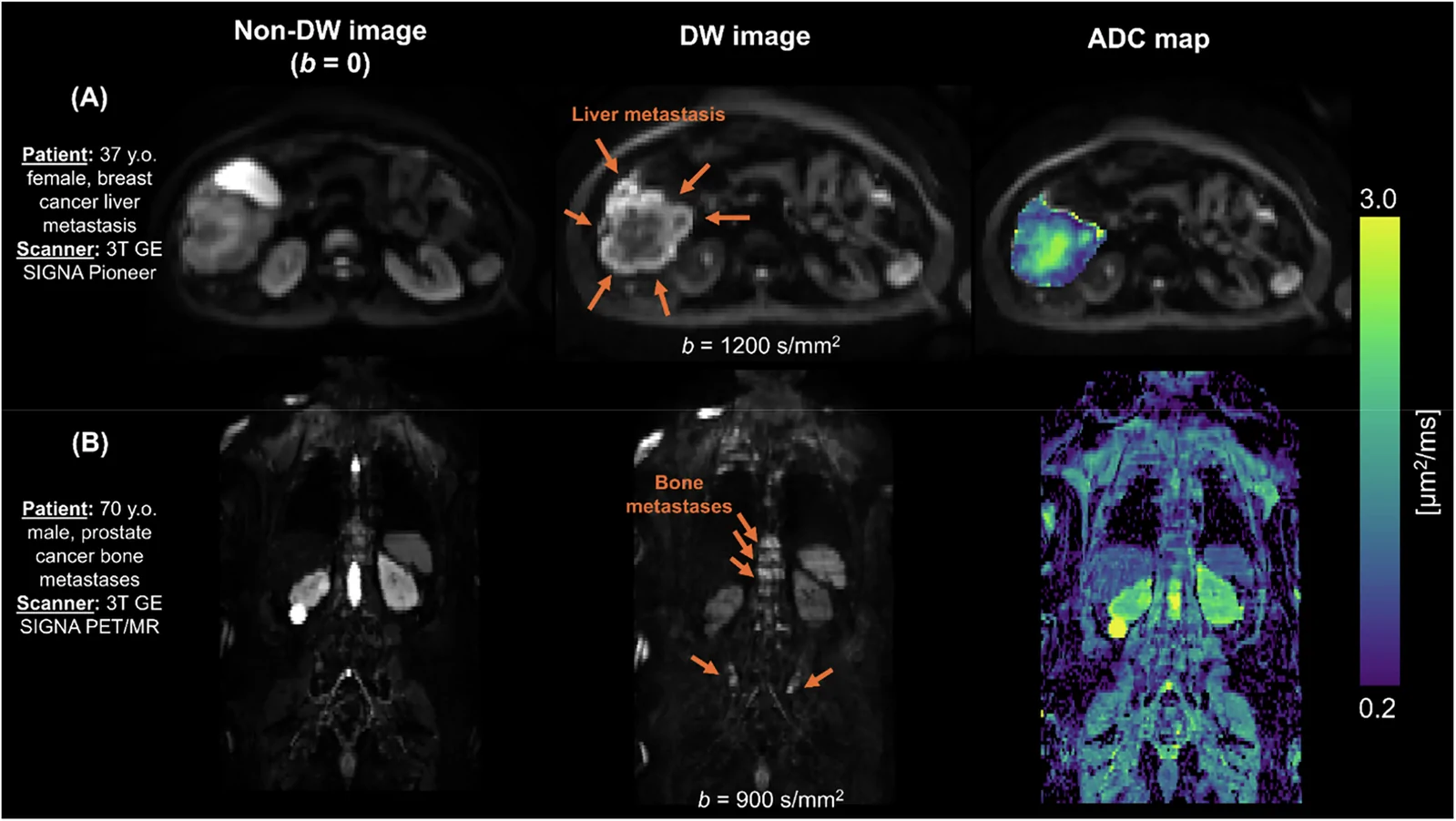
Examples of DW images and ADC maps in body tumours. From left to right: non-DW image (*b* = 0); DW image; ADC map. **A** 37-year-old breast cancer female patient, presenting with a liver metastasis, was scanned on a 3 T GE SIGNA Pioneer system. **B** 70-year-old prostate cancer male patient, presenting with disseminated bone metastases. The figure demonstrates the unique ability of DWI to highlight between- and within-tumour contrasts, e.g., intense DW signal attenuation (and hence high ADC) in the core of the breast cancer liver metastasis, compatible with the presence of a tumour necrotic core. Images courtesy of Raquel Perez-Lopez, Francesco Grussu, Alonso Garcia-Ruiz (VHIO, Barcelona, Spain)

DWI can also be acquired as whole-body imaging within clinically acceptable scan times, making it a feasible and well-tolerated option for patients. We recommend whole-body DWI for screening high-risk populations, evaluating the extent of disease, and assessing treatment response through tracking ADC changes, especially in cancers affecting the skeleton, e.g., prostate cancer with bone metastases [19] or multiple myeloma [20]. To enhance tumour detectability, we recommend DWI with background body signal suppression (DWIBS) (Fig. 6). However, quantitatively, DWIBS may lead to misestimation of ADC values [21].

A summary of DWI recommendations in radiology is in Table 1.

**Table 1:** Summary of the recommended protocols and clinical utility of DWI in radiological applications

Clinical Field	Protocol Recommendations	Key Diagnostic Utility
General DWI	• Single-shot EPI standard • TR ≥ 4500 ms (minimise T1) • Minimum TE for target *b*-value • Monopolar (for better SNR) or bipolar (for better eddy currents compensation) • 3–4 directions (routine) or ≥ 6 (DTI for highly anisotropic tissues) • Fat suppression when necessary	• ADC maps • 4-scan trace image • Calculated high *b*-value images (possible for enhanced contrast-to-noise-ratio)
Neuro	• *b* = 0 + *b* = 1000 s/mm^2^ standard • Non-EPI for skull base	• Stroke: ADC reduction in acute, pseudo-normalisation at ~1 week • Abscess vs tumour differentiation • Correct T2-shine-through with ADC (e.g., in subacute cerebral infarction)
Head and Neck	• *b* = 50 s/mm^2^ + *b* = 800–1000 s/mm^2^ • Non-EPI for cholesteatoma • Fat suppression	• Cholesteatoma detection • Lymph node characterisation • Reduces second-look surgeries
Body/oncology	• *b* = 50 s/mm^2^ + *b* = 800-1000 s/mm^2^ (*b* = 1500 s/mm^2^ optional) • Respiratory gating for liver • DWIBS for whole-body	• Prostate: PI-RADS (PZ dominance) • Breast: benign vs malignant • Liver: LI-RADS ancillary feature • Bone metastases detection

## Beyond the ADC

**Tractography** maps white matter fibres but requires a large number of directions (≥ 30; e.g. using high-angular resolution diffusion imaging (HARDI) [22]), resulting in long acquisition times. It is clinically recommended for neurosurgical planning of tumour resection, epilepsy surgery, and deep brain stimulation [23]. Challenges such as crossing fibres, sharp fibre angles, and oedema can be handled by advanced tractography software, leading to more comprehensive tractograms, at the expense of false positives [24].

**Diffusion Kurtosis Imaging (DKI)** provides information about the heterogeneity of ADCs within the voxel and is thus complementary to the overall ADC [25]. This so-called diffusional kurtosis parameter can be mapped from DWI performed with at least three *b*-values (e.g. *b* = 0, 1000, 2000 s/mm²) (Fig. 3C). DKI’s clinical value over conventional DWI has been demonstrated in brain tumours, neurological diseases, stroke, injury, and prostate/liver lesions [26], though there is no explicit clinical recommendation yet.

**IntraVoxel Incoherent Motion (IVIM) **models blood flow in capillaries as pseudo-diffusion with a coefficient D*, mainly affecting low *b*-values (< 200 s/mm^2^) [27]. IVIM influences ADC and its effect, if undesired, can be removed by excluding low *b*-value signals. The perfusion and genuine diffusion contributions can also be separated, which enables perfusion mapping (blood volume fraction *f* and D*) without contrast agents (Fig. 3D) [28]. IVIM aids oncology by mapping tumour vascularisation, but is not yet routine in clinical practice.

**Biophysical models.** ADC and DKI lack compartment-specific details, which advanced biophysical models [29, 30, 31] offer, promising improved tissue characterisation, early and more specific diagnosis, and enhanced understanding of pathophysiological processes, but at the cost of longer scans and complex processing.

## Summary statement

DWI has evolved into a critical tool across multiple radiological applications, offering valuable insights into tissue microstructure and pathology. Its potential is expected to increase further with the implementation of standardised protocols and advanced methods to enhance image quality and provide more specific information on tissue content.

However, its successful implementation requires careful consideration of acquisition protocols, adherence to standardised guidelines, and awareness of potential artefacts and pitfalls in image interpretation. Optimising DWI protocols for specific clinical applications, ensuring inter-scanner consistency, and integrating DWI seamlessly into clinical workflows will enhance diagnostic accuracy and reproducibility. Moreover, rigorous quality control measures and expert interpretation are essential to mitigate artefacts and improve confidence in findings.

By following the recommendations and guidelines summarised in this work from experts of the ESMRMB, radiologists and imaging professionals can maximise the clinical utility of DWI, ultimately improving patient care and outcomes.

## Patient summary

DWI is a non-invasive MRI technique that provides unique insights into tissue structure and content at the cellular level in various organs. It is widely used for clinical applications in the brain and the body, for neurological disorders (e.g., stroke detection and assessment), and for cancer diagnosis and monitoring.

This paper summarises recommendations and guidelines to ensure consistent and accurate DWI results across different hospitals and scanners. Since DWI findings require expertise to interpret, we provide guidelines to reduce variability and improve diagnostic confidence. These guidelines also help ensure DWI is used appropriately, maximising its benefits for patients.

## Abbreviations

ADC: Absolute diffusion coefficient
DTI: Diffusion tensor imaging
DWI: Diffusion-weighted imaging
EPI: Echo-planar imaging
SNR: Signal-to-noise ratio
TE: Echo time
TR: Repetition time
